# Organizational Factors in Clinical Data Sharing for Artificial Intelligence in Health Care

**DOI:** 10.1001/jamanetworkopen.2023.48422

**Published:** 2023-12-19

**Authors:** Alaa Youssef, Madelena Y. Ng, Jin Long, Tina Hernandez-Boussard, Nigam Shah, Adam Miner, David Larson, Curtis P. Langlotz

**Affiliations:** 1 Department of Radiology, Stanford University School of Medicine, Stanford, California; 2 Department of Medicine, Biomedical Informatics Research, Stanford University School of Medicine, California; 3 Department of Pediatrics, Stanford University School of Medicine, Stanford, California; 4 Department of Biomedical Data Science, Stanford University School of Medicine, Stanford, California; 5 Department of Psychiatry, Stanford University School of Medicine, Stanford, California

## Abstract

**Importance:**

Limited sharing of data sets that accurately represent disease and patient diversity limits the generalizability of artificial intelligence (AI) algorithms in health care.

**Objective:**

To explore the factors associated with organizational motivation to share health data for AI development.

**Design, Setting, and Participants:**

This qualitative study investigated organizational readiness for sharing health data across the academic, governmental, nonprofit, and private sectors. Using a multiple case studies approach, 27 semistructured interviews were conducted with leaders in data-sharing roles from August 29, 2022, to January 9, 2023. The interviews were conducted in the English language using a video conferencing platform. Using a purposive and nonprobabilistic sampling strategy, 78 individuals across 52 unique organizations were identified. Of these, 35 participants were enrolled. Participant recruitment concluded after 27 interviews, as theoretical saturation was reached and no additional themes emerged.

**Main Outcome and Measure:**

Concepts defining organizational readiness for data sharing and the association between data-sharing factors and organizational behavior were mapped through iterative qualitative analysis to establish a framework defining organizational readiness for sharing clinical data for AI development.

**Results:**

Interviews included 27 leaders from 18 organizations (academia: 10, government: 7, nonprofit: 8, and private: 2). Organizational readiness for data sharing centered around 2 main constructs: motivation and capabilities. Motivation related to the alignment of an organization’s values with data-sharing priorities and was associated with its engagement in data-sharing efforts. However, organizational motivation could be modulated by extrinsic incentives for financial or reputational gains. Organizational capabilities comprised infrastructure, people, expertise, and access to data. Cross-sector collaboration was a key strategy to mitigate barriers to access health data.

**Conclusions and Relevance:**

This qualitative study identified sector-specific factors that may affect the data-sharing behaviors of health organizations. External incentives may bolster cross-sector collaborations by helping overcome barriers to accessing health data for AI development. The findings suggest that tailored incentives may boost organizational motivation and facilitate sustainable flow of health data for AI development.

## Key Points

**Question:**

Are organizational factors associated with the motivation of health organizations to share clinical data for artificial intelligence (AI) development?

**Findings:**

In this qualitative study, 27 leaders from 18 health organizations were interviewed, and a predominant concern among them was data privacy risks. Most stakeholders viewed these as a substantial barrier for public health data sharing due to potential liability and reputational consequences; however, they identified external incentives as key factors for enhancing organizational motivation and fostering both within and across-sector data-sharing collaborations for AI development.

**Meaning:**

The findings of this study suggest that data-sharing policies should be rooted in feasibility and incentivization strategies to promote responsible and equitable AI development in the health care sector.

## Teaser

This qualitative study examines issues identified by representatives from different health sector organizations on the development of artificial intelligence and sharing of health data.

## Introduction

Advances in algorithm development have shown that artificial intelligence (AI) and machine learning can augment clinical decision-making, promoting diagnostic excellence.^1,2,3,4^ While there is consensus that data flow between organizations and developers is necessary to develop AI algorithms, many organizations remain reluctant to share health data for AI due to organizational apprehensions and competing priorities, affecting equitable AI development in the health care sector.^5,6,7,8,9^

Current research highlights problematic trends in health data sharing. A systematic review by Kaushal et al^10^ highlighted that large data-sharing initiatives are often performed by large academic institutions from a few geographic locations with access to funding and the technical expertise to transform and share well-curated health data sets responsibly. While these data sets have been foundational for AI development, they often lack comprehensive representation across geographic, racial, and socioeconomic dimensions. This underrepresentation in health data sets precludes effectively generalizing algorithms to the wider population.^1,9^

Although previous studies^11,12^ have acknowledged the presence of organizational factors, there remains a gap in understanding how these factors specifically impact organizational readiness for sharing health data for AI development. While insights from the biomedical and genetic realms have highlighted challenges, such as data standardization, ethical dilemmas, and reidentification risks, AI introduces additional barriers to health data sharing that remain unexplored.^13,14^ Thus, studying the organizational factors affecting the flow of health data in the health care sector is crucial to the future of AI development.

To address this knowledge gap, we designed a qualitative case-based study to explore health data-sharing behavior across 18 organizations in the academia, government, nonprofit, and private health sectors. Our primary study objectives addressed (1) factors that motivate data sharing in health organizations, (2) organizational factors facilitating or hindering data-sharing efforts, (3) how the organizational structure (by sector) relates to motivation for clinical data sharing, and (4) factors that affect cross-sector data-sharing collaboration.

## Methods

This qualitative study was approved by the Stanford School of Medicine Institutional Review Board. All participants read waiver of consent forms and provided verbal consent to be interviewed; no financial compensation was provided. This study followed the Consolidated Criteria for Reporting Qualitative Research (COREQ) reporting guideline for qualitative research.

### Study Design, Participants, and Recruitment

**Figure 1.**
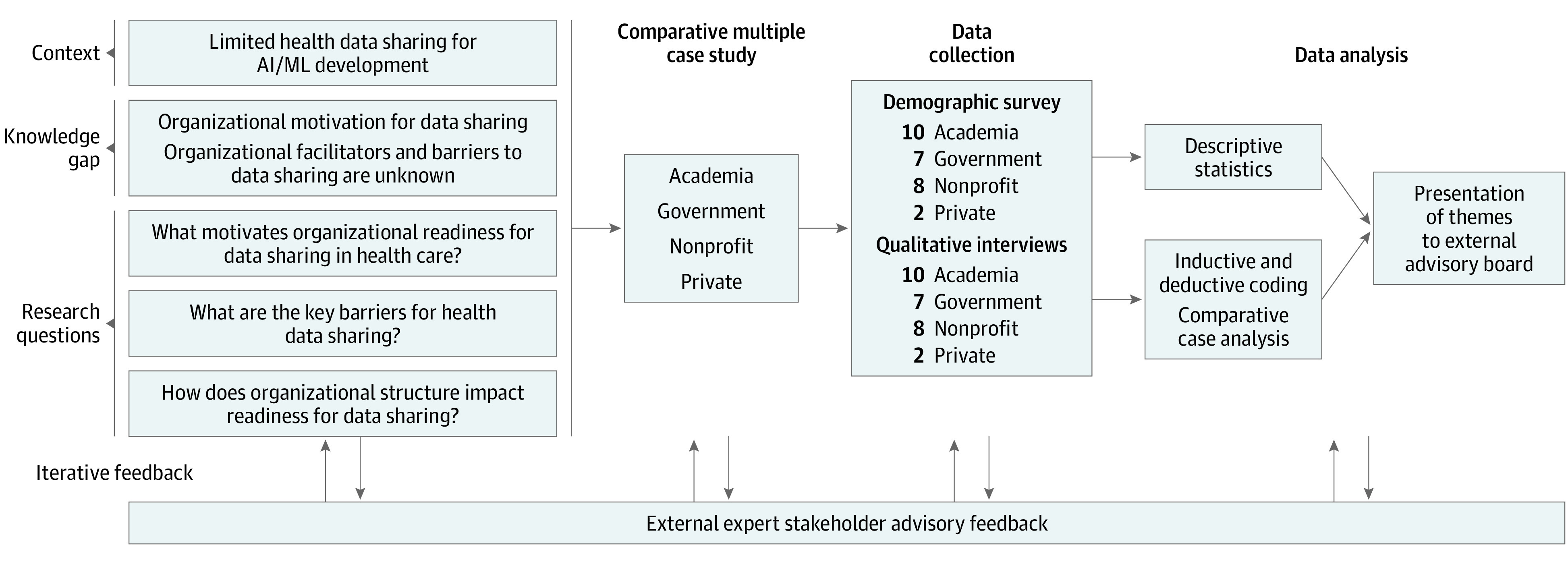
Overview of Study Design AI indicates artificial intelligence; ML, machine learning.

This study used a qualitative case-based design, an embedded multiple case study, to investigate organizational factors affecting data-sharing behaviors of organizations across 4 sectors (academia, government, nonprofit, and private)^15,16^ (Figure 1). The academic sector included academic health centers. Government organizations comprised foundations and funding agencies that engage with health data, overseeing and funding health data-sharing initiatives, the nonprofit sector included data brokers and developers engaged in data sharing for public good, and the private sector included data brokers and developers engaged in data sharing for monetary gains. The selection of sectors and organizations for inclusion in this study was informed by a comprehensive literature review that identified entities actively involved in AI development or possessing the ability to share health-related data. Invitations were extended to individuals in leadership roles guiding data-sharing initiatives in their respective organizations.

A purposive, nonprobabilistic sampling strategy was used to maximize organization diversity and identify 78 individuals from 52 health organizations.^17^ Individuals who agreed to participate in this study were invited to complete an in-depth semistructured interview conducted in the English language (August 29, 2022, to January 9, 2023). All interviews were recorded with participant permission for publication of findings and quotations and transcribed verbatim by a professional transcription service (Athreon). According to constructivist-grounded theory techniques, interview sampling was iterative and informed by parallel data analysis that continued until additional interviews did not yield novel data or nuances to the emerging themes, indicating theoretical saturation at both the conceptual and thematic levels.^18^

During the interview, participants completed a brief demographic survey that captured the participant’s role and organization’s data-sharing activity (eAppendix in Supplement 1). This information guided the content and direction of the discussions in the semistructured interviews. Interviews were facilitated using an interview guide that the research team (A.Y., M.Y.N., J.L., A.M., D.L., and C.P.L.) iteratively developed (eAppendix in Supplement 1). The interview guide was further refined based on feedback from an external advisory group (identified in the Additional Contributions section) comprising experts in machine learning development, data set sharing, clinical informatics, data harmonization, curation, AI bias, organizational leadership, ethics, and qualitative research. All interviews were led by a researcher with more than 7 years’ experience in qualitative AI health research (A.Y.). Interviews lasted between 30 and 60 minutes and were conducted in English through Zoom (Zoom Video Communications). In each interview, participants provided insights into their positions and an overview of the data-sharing procedures of their respective organizations. Subsequent open-ended and probing questions encouraged them to elaborate on their motivations for engaging in data-sharing efforts, identify factors that facilitated or hindered such efforts, and discuss the outcomes observed with organizational norms and policies regarding organizational data-sharing decisions.

### Data Analysis

Quantitative data from these surveys were analyzed using Microsoft Excel (Microsoft Corporation, version 16.78.3) to compute descriptive statistics. Qualitative interview transcripts were analyzed from a constructivist paradigm, informed by the constructivist-grounded theory approach, allowing for interpretive understandings and iterative logic to question the data and advance conceptual development. First, the interview transcripts were inductively coded by one of us (A.Y.) to generate a preliminary codebook. This codebook was subsequently used by the study members (M.Y.N. and additional contributor D.S.) who independently deductively coded the interview transcripts to validate coding credibility. The study team (A.Y., M.Y.N., and D.S.) met and reached consensus on any coding disagreements—a process that achieved more than 80% agreement.

As the study advanced, one of us (A.Y.) continued engaging in open and axial coding processes to identify emerging constructs and confirm theoretical saturation.^18,19,20^ The entire team (A.Y., M.Y.N., J.L., T.H.B., A.M., D.L., and C.P.L.) met on a weekly basis to deliberate on developing conceptual categories, ensuring the triangulation of themes by drawing from their diverse expertise in machine learning, organizational leadership, data sharing, clinical informatics, and qualitative research. These collaborative discussions contributed to conceptual refinement, ensuring that the emerging themes were both representative of the participants’ perspectives and grounded in the data. All qualitative data were coded using analytical software (AXQDA Analytics Pro, Release 22.2.0). Throughout the research, one of us (A.Y.) engaged in memo-writing and maintained an audit trail of analytical decisions to promote reflexivity and transparency.

### Conceptual Framework and Theory Development

A conceptual framework that explains how specific organizational factors may be associated with data-sharing behavior was developed through inductive and deductive approaches in 4 phases: (1) identify the generalizable key factors explaining data sharing, (2) generate and refine emerging hypotheses through data collection and analysis, (3) map the variables and outcomes using the abstraction hierarchical approach, and (4) triangulate the emerging framework through iterative team analysis and establish organizational theories in health care.^11,12,21^ This framework is considered a determinant framework through the implementation science typology.^22^

## Results

### Framework for Organizational Readiness for Data Sharing

**Figure 2.**
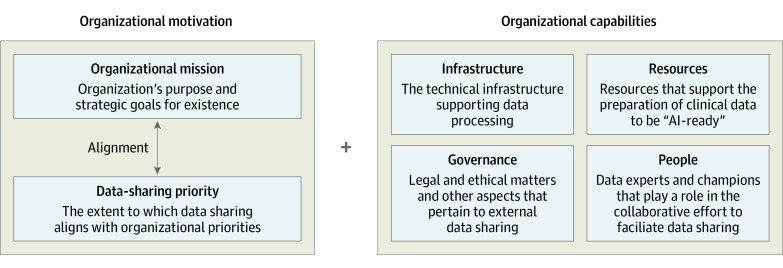
Organization Readiness for Sharing Artificial Intelligence (AI)-Ready Clinical Data Organizational readiness is composed of 2 elements: organizational motivation and capabilities. For organizational motivation to exist, alignment in data-sharing and organizational priorities is key. Organizational capabilities refer to the facilitators that enable organizations to share data, including technical infrastructure, resources, people, and governance. Incentives can sway organizational motivation for data sharing (not shown for simplicity).

We interviewed 27 organizational leaders from 18 organizations, including 10 (37.0%) from academia, 7 (25.9%) from government, 8 (29.6%) from nonprofit organizations, and 2 (7.4%) from private organizations (Table). Most of the participants (24 of 27 [88.9%]) were US-based. The framework for organizational readiness comprised 2 constructs from stakeholder perspectives: organizational motivation and organizational capabilities (Figure 2).

**Table.** Demographic Characteristics and Breakdown of the 18 Organizations and 27 Leaders Interviewed

Sector	No. of organizations (%)	No. of interviews (%)	Leadership roles	Types of data shared	Role in the data ecosystem	Geographic location
Academia	6 (33.3)	10 (37.0)	Clinical data science leaders, chief compliance officer, chief informatics officer, chief innovation officer, chief operation officer, executive director, data integration managers, data scientists	Imaging, genomics, EHR, clinical reports	Data acquirer, data aggregator, data curator, data sharer, data user, data validator	US
Government	4 (22.2)	7 (25.9)	Head of research, chief executive officer, chief research information officer, program directors, branch chief technology officer	Imaging, EHR	Data curator, data sharer	US, UK
Nonprofit	6 (33.3)	8 (29.6)	Vice president, chief data scientist, ethics data policy analyst	Imaging, EHR	Data curator, data sharer, data validator	US
Private	2 (11.1)	2 (7.4)	Chief executive officer, research scientist	Imaging, EHR	Data curator, data user, data sharer	US

Abbreviation: EHR, electronic health record.

#### Organizational Motivation

Organizational motivation refers to the degree to which data sharing and its perceived value align with organizational values, missions, and priorities. Although organizational motivation is a crucial aspect of organizational readiness to engage in data-sharing initiatives, it can greatly vary between sectors. For example, some organizations may be willing to support data-sharing efforts, even without direct financial gains, if they align with their mission. However, private organizations may be more motivated by financial gains and remaining competitive in the data-sharing market.

“Government organizations and academia ultimately have the good of the public in mind…we are meant to be a neutral entity that evaluates science and medicine in a way that isn’t biased by profit. The principles of our mission and nonprofit status really do guide us here. That’s why we can put governance in place that prioritizes ethics over profit” (academia, chief operating officer).

“As a company, we have pressure from our venture capitalists—our investors say you need to make money….” (private, chief executive officer).

#### Organizational Capabilities

The second theme centers around organizational capabilities, or the infrastructure, resources, people, and data-sharing governance required to develop and ensure responsible data sharing. In other words, this theme is rooted in whether an organization has the capacity to engage in and oversee data sharing, because transforming clinical data to be AI ready is complex, especially when data aggregated from multiple sites have disparate data formats and content.

“If you’ve got dozens and dozens of sites that are streaming data into you, then you need to have people that are going to manage that data and organize it as it’s coming in. It’s a huge effort…and everybody has a certain level of expertise for doing each piece of it” (government, data curation expert).

Interviewees described that, despite willingness to participate in data-sharing efforts, the appropriate infrastructure and people to both manage and process the data are key requirements. Clinical experts are critical to ensuring appropriate context and quality for data to be meaningful for AI research.

“...It requires background contextual knowledge…. For every single variable that you’re going to curate out of EHR [electronic health record] data into a feature table or any sort of downstream transformation, that contextual knowledge is absolutely key in order to make sure that what you get downstream isn’t a total misrepresentation of what really happens with a patient” (government, data informatics expert).

Another dimension is the regulation surrounding data sharing. The authorization process for sharing data is arduous and considers the distinct use case of the data, the data type, and contextual factors. Moreover, given the various risks and consequences of data deidentification and inappropriate handling, organizations tend to incorporate a variety of safeguards to ensure data privacy.

“There are a lot of agreements in place when it comes to institutional sharing….If it is private funded, sponsor funded, sponsored research, again, there’s going to be contractual obligations between the sponsor and the participating institutions. The federal government also oversees, they want to make sure if it is human subjects research, there are regulations that the investigators, the institutions have to meet and be in compliance, otherwise there’s fines and penalties. Just like if HIPAA [Health Insurance Portability and Accountability Act] is violated” (academia, regulatory and compliance officer).

However, organizations are not monolithic, but rather comprise individuals with diverse capabilities and motivations. In some instances, individual champions can strongly shape their organization’s data-sharing activities. Particularly in academia and government, champions can help organizations bridge intraorganizational and interorganizational data-sharing silos and overcome some associated hurdles, stimulating an organization’s capability for data sharing, especially when the effort aligns with organizational values and goals.

“[One] of the main components that makes the partnership successful is having a couple different internal champions. Someone internal to the partner organization who is excited about what [the organization] is doing and wants to see the data shared to either further their own research or to help inform a problem that the organization or the person is really invested in. Having those internal champions means there’s just a better success rate of navigating through all the tons of bureaucratic hurdles….” (nonprofit, executive director).

### Data-Sharing Behavior by Sector

**Figure 3.**
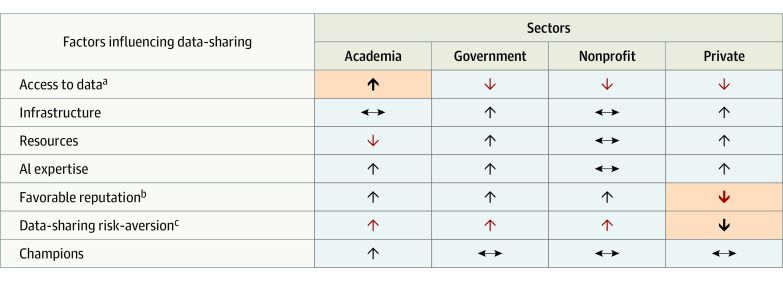
Strengths and Weaknesses in Data-Sharing Factors Across Academia, Government, Nonprofit, and Private Sectors The black upward arrowheads symbolize increase (facilitator) whereas red arrowheads represent barrier associated with decreased data sharing. Sideways arrowheads represent a variable factor. Shaded cells distinguish unique strengths or challenges for a sector compared with the others. ^a^Academia has distinct access to health data. ^b^Private sector faces challenges in public data-sharing collaborations. ^c^Private sector is notably less risk averse to data-sharing compared with the other 3 sectors.

Within the above-described framework, we found that sector-specific differences related to the greater data-sharing ecosystem that comprises these entities (Figure 3). For example, a key capability of academic medical centers is accessibility to relevant clinical data, along with other capabilities that promote data-sharing champions, garner public trust, and attract extramural funding. A strength of government organizations is the ability to create incentives for other organizations to share data, helping align an organization’s mission with its prioritization for data sharing.

“What makes an institution share data when they wouldn’t ordinarily share it? Sometimes it’s just other peer institutions who are sharing when they’re not. If they feel as though they’re going to miss out on important research, that can be an incentive” (data privacy expert, academia).

These collaborations are often bolstered by nonprofit and private intermediaries serving as trusted data brokers, facilitating access to deidentified ealth data that complies with privacy laws. For example, private organizations have begun to enable large-scale data sharing by providing cloud storage and environments that improve access to AI-ready health data.

“Industry originally helped us design our platform…. We didn’t want to reinvent the wheel. Why would you reinvent the wheel when there’s already security and other great features that we could make HIPAA-compliant, FISMA [Federal Information Security Management Act]-low certified as well. We improved the security, and they provided us with research credits because we’re a large organization” (nonprofit, data science lead).

Nevertheless, organizations within the academic sector often display risk-averse tendencies to sharing data, primarily due to reputational and public trust concerns. To minimize these risks, these organizations tend to favor a conservative approach to data deidentification, which incurs cost and labor, making data sharing burdensome.

“[Patients have] entrusted us with that data to make decisions regarding their care, and we want to make sure that again, with our collaborations, whether it is research related or we’re doing something to advance knowledge, to work with one of our business partners…. We want to have assurances that while we’re in that partnership or that collaboration, if we were to choose to go with another vendor… we have a way to get our data back” (academia, regulatory privacy expert).

Similarly, although academic and government organizations aim to promote public good through scientific discovery and even if they are motivated to share data, how much each organization engages with such efforts can be associated with additional extrinsic incentives to make this process sustainable. These incentives, encompassing both financial and reputation benefits, can catalyze data-sharing behavior in organizations otherwise unmotivated to participate in such efforts.

“I’m all for the public good. I love the public good, but I think we need to define public good in ways that are sharper. Like, if you have specific costs attached to making data available, you can’t just say, believe in public good (capital P and capital G). That’s not going to work” (academic and nonprofit, organizational leader).

To fulfill their missions, academic and government organizations have developed a symbiotic relationship based on their complementary capabilities and aligned motivations. For example, government organizations rely on academic institutions to contribute valuable data to their repositories, while academic institutions capitalize on government funding to build the technical infrastructure required to enable responsible data sharing across multi-institutional systems. Such relationships are a key accelerator of public and research network collaborations across these sectors.

Cross-sector collaborations are also essential for nonprofit and private organizations. However, a common barrier here is building trustworthy relationships with academic medical centers and integrated health systems (Figure 3). Because academic medical centers have higher standards for data stewardship, private organizations struggle to establish data-sharing agreements, particularly with large academic medical centers. This is where nonprofit organizations can act as data intermediaries. For example, professional societies, which fall under the umbrella of nonprofit organizations, can act as trusted data brokers facilitating data-sharing partnerships among the academic, government, and private sectors.

Overall, organizational readiness across sectors is dependent on the unique capabilities of each organization and the extent to which extrinsic incentives can stimulate organizational motivation by aligning data sharing with the organization’s mission.

## Discussion

The flow of health data between data providers and users is essential for AI development. However, the factors affecting data-sharing behavior have not been extensively studied. To address this gap, we conducted a qualitative cross-sectional study of factors shaping data-sharing behaviors in the academic, government, nonprofit, and private sectors. This investigation generated 3 key findings with implications for accelerating responsible data sharing for AI in health care.

First, organizational motivation and capabilities are the core factors affecting organizational behavior surrounding data sharing. Although previous studies have proposed alternative approaches and frameworks that attempt to relate the involved parties and stakeholders,^6^ they have mostly focused on specific factors, without elucidating the dynamic interactions among the identified factors. Our conceptual framework more broadly categorizes organizational readiness for data sharing into organizational motivation and capabilities before further exploring how these constructs affect each organization’s behavior and role in the data-sharing ecosystem. Recognizing and differentiating the components within these constructs is paramount to addressing the various barriers impacting organizations’ readiness for clinical data sharing.

Second, our cross-sector analysis found that organizational capabilities surrounding data sharing varied greatly across sectors. While government, nonprofit, and private organizations exhibited greater resource availability, academic organizations held distinct advantages in access to health data, granting them more control on data-sharing agreements and data availability as a whole. Because of this variation, collaborations across sectors are symbiotic, helping to compensate for sector-specific barriers and representing a key approach to ensuring equitable data sharing and more generalizable AI algorithms.^10^

Third, in-depth understanding of organizational structure and roles enabled us to identify the diverse approaches of each sector to the regulations surrounding data sharing. For example, the conservative approach of academic organizations stemmed from the necessity for increased compliance and mitigation strategies to safeguard data privacy and ensure proper use of the shared data. Interviews with stakeholders showed data ownership and stewardship to be major concerns, often stifling data-sharing partnerships, with privacy issues being the primary concern. Thus, collaborations both within and across sectors were more likely to be successful if the organizations involved demonstrate aligned values and complementary capabilities to overcome regulatory hurdles.

Overall, the degree to which an organization’s values align with its prioritization of data sharing appears to define its motivation to engage in data-sharing efforts (Figure 2). However, organizations with a low degree of alignment, and thus low motivation, can be persuaded by extrinsic financial or reputational incentives. For example, extramural funding from government organizations can compel academic institutions to participate in data-sharing collaborations, and private organizations may agree to partnerships with intermediary nonprofit data brokers to bolster their public perception.

### Limitations

This study has limitations. Despite extensive efforts to reach private data brokers and integrated health systems, the response from these sectors was low, which may alter the overarching narratives on data sharing. By focusing on organizations already engaged in data sharing, this study may have inherent limitations in capturing the broader challenges and reservations faced by organizations not represented in this study. Additionally, most (88.9%) of the studied organizations were US based, potentially limiting the generalizability of our findings to non-US organizations, particularly given differences in data privacy regulations.

This exploratory study has shed light on the factors affecting the readiness of organizations for health data sharing for AI development. The study offers a plausible explanation to recognize the relationship between organizational motivation, capabilities, and context. However, there remains a gap in understanding private health care organizations possibly less inclined toward data sharing to shed light on the organizational barriers from this perspective. Future research should explore how extrinsic incentives can boost organizational motivation and address competing priorities that stifle cross-sector data-sharing collaborations. Addressing these barriers will be pivotal to ensure a sustainable flow of health data through the ecosystem to enhance AI development in the health care sector.

## Conclusions

Our research suggests that an organization’s readiness for health data sharing for AI development is determined by its motivation and capabilities. We identified that sector-specific factors play a pivotal role in organizations’ data-sharing behavior and that extrinsic incentivization can substantially bolster collaborations to overcome these sector-specific barriers. To improve the sharing of health data, aligning health data sharing policies with incentives that motivate organizations is crucial. This alignment may help increase the sharing of health data in a way that is sustainable, responsible, and fair.

## Notes

Supplement 1. **eAppendix.** Quantitative Demographic Survey

Supplement 2.
Data Sharing Statement
