# Supplementary material for: Organizational Factors in Clinical Data Sharing for Artificial Intelligence in Health Care
Source: JAMA Netw Open. 2023 Dec 19;6(12):e2348422. doi: 10.1001/jamanetworkopen.2023.48422 (PMC10731479; doi:10.1001/jamanetworkopen.2023.48422)
Supplement: Supplement 1. — eAppendix. Quantitative Demographic Survey [file jamanetwopen-e2348422-s001.pdf]

# Supplementary Online Content

Youssef A, Ng MY, Long J, et al. Organizational factors influencing clinical data sharing for artificial intelligence in health care. *JAMA Netw Open*. 2023;6(12):e2348422. doi:10.1001/jamanetworkopen.2023.48422

**eAppendix.** Quantitative Demographic Survey

This supplementary material has been provided by the authors to give readers additional information about their work.

© 2023 Youssef A, et al. *JAMA Network Open.*

**eAppendix: Quantitative Demographic Survey**

Demographic Questions

- Race/ethnicity
- Age
- Sex
- State
- Country

Current role

Which of the following positions best describes your current role? (please check all that apply)

- Ethics Officer: defines and develops ethical standards that support safe and responsible data-sharing at organization.
- Compliance/Regulatory Officer: Oversees ethical and legal compliance within the organization, and ensures compliance with laws, regulatory requirements, policies, and data management procedures.
- Privacy Officer: Evaluates organization health data de-identification policies and procedures for research in compliance with HIPAA.
- Dataset Creator: Person responsible for the creation of the dataset used for AI health applications.
- Machine Learning Researcher: Person responsible for the use of the dataset for the development of AI models for health applications
- Other (please describe)

Sub-roles

Given your position, what specific role did you play in the public data creation/sharing/dissemination process?

- Data acquisition - played a role in consolidation self-reported, EHR, sensors/wearable, genetic data, or other data relevant to the dataset
- Data de-identification - played a role in the data de-identification process
- Data curation - played a role in aggregating, selecting, organizing, harmonization, and managing data to meet the needs of researchers or other stakeholders
- Data storage - played a role in identifying an appropriate storage space for the data
- Data annotation - played a role in the modification of the data to contain unique research information
- Data documentation - played a role in process of recording any aspect of project design, sampling, data collection, cleaning and analysis that may affect results
- Data analysis/usage to create AI models - played a role in using the dataset data to create AI models for healthcare applications
- Obtain IRB approval to use and share the dataset - played a role in obtaining institutional approval (e.g., IRB) to use and share the dataset
- Data-sharing authorization (organization-level) - authorizes the public release of health data for machine learning (ML) or artificial intelligence (AI) research and/or application development

© 2023 Youssef A, et al. *JAMA Network Open.*

- Sharing/dissemination of the dataset (individual-level) - played a role in sharing/disseminating the dataset
- Dataset management (individual-level) - played a role in the continuous management of the dataset
- Dataset management (organization-level) - monitors the management of clinical databases for AI research and applications development across the organization

<u>Sub-questions if "Ethics Officer", "Compliance/Regulatory Officer", or "Privacy Officer" selected as current role:</u>
How long have you been in this role?

Sub-question: If less than 2 years in the current role, please indicate the former position ………..

<u>Organization Context</u>
Sectors
Which of the following sectors best represent this organization?
- Academia
- Government
- Private sector (i.e., industry)
- Non-profit (i.e., professional society, collaborative networks)
- Other

Which of the following roles apply to your organization: (please check all that apply)
- Data acquirer: collects health data for research given patient consent for responsible use of clinical data for the improvement of care. Health data may include: electronic health record (EHR),genomics, blood samples, images, wearable devices, biospecimens, patient reported-surveys, sociodemographic.
- Data Curator: assembles, aggregates, structures data in a harmonized format that is machinable for AI research or applications development.
- Data Public Releaser: promotes public release of health data for machine learning/ artificial intelligence use.
- AI Validator: validates the performance of built-in or purchased AI-solutions.
- AI Application Developer: Utilizes health data for research to develop machine learning decision support tools to improve care quality.
- AI Application Consumer: Uses or purchases AI-based applications to improve care delivery.
- AI Applications Beneficiaries: derives benefit from the use of AI-based applications in care delivery.

© 2023 Youssef A, et al. *JAMA Network Open.*

## Appendix B: Semi-structured Key Informant Interview Questionnaire

1. Facilitators and Barriers for Data-Sharing
    a. What motivates regular data-sharing at this organization with the public or other organizations in the healthcare ecosystem?
    b. What were the incentives that promoted data-sharing at the institution?
    c. What are some barriers for curating and releasing health data for AI research?
    d. What were the main barrier(s) to overcome to enable data-sharing?
    e. Which entities at your organization are involved in the data-sharing process?

2. Nature of the Data-Sharing
    a. Describe the type(s) of data that are shared at this institution.
    b. What major facilitators led to the success of your data sharing strategy?
    c. Do you share data with profit, no-profit, or both? Why?
    d. Who can access datasets generated by your organization?
    e. What types of datasets may have restricted or controlled access? Why?

3. Ethics and Regulatory Compliance
    a. What ethical framework or standards guide the development of data-sharing policy at this organization?
    b. Why was this ethical framework chosen? (stakeholders' values and norms, accountability, safety etc)
    c. What role does your institutional review board(IRB) play in the data-sharing process?
    d. What is the process to determine ethical and regulatory compliance and approve a data-sharing request?
    e. What are common ethical or regulatory obstacles that can slow down data-sharing approvals? How are these challenges addressed?
    f. Datasets differ in access level depending on data types. Does the ethics and compliance approval process differ if public vs. restricted, vs.control?
    g. What are the required compliance criteria to approve public release of health datasets for ML at your institution?
    h. Who are the stakeholders involved in the development of the data user agreement at your institution?
    i. Does your organization restrict dataset use for research only or allow for commercial use? Why or Why not?
    j. If a patient withdraws their consent for research after publicly releasing a dataset including patient de-identified clinical data, what are some steps the ethics and compliance office might pursue?
    k. What is the process to ensure organization compliance with external regulations/bylaws relevant for publicly sharing health data?

4. Data Privacy & Risk Management

© 2023 Youssef A, et al. *JAMA Network Open.*

a. What are the technical factors that might hinder the data-sharing process at your organization?
b. Data de-identification is a complex and iterative process, what are the criteria to assess data de-identification plans and re-identification risks?
c. Data de-identification is the removal or alteration of information in a dataset to protect privacy by reducing the ability to associate data with specific individuals. There are numerous ways to de-identify data each with different results and levels of effectiveness. How do you determine data de-identification approaches are effective to mitigate privacy risks?
d. The process to prepare, curate, and share data can be expensive. What are some successful financial strategies to enable periodic data-sharing at your institution?

Additional Questions:

a. What are the *conditions* under which organization might choose to share—*or not share with the public*?
b. From your perspective/role, what makes a healthcare organization ready (has the capacity to use and share health data for AI)?
c. What are the main barriers for health care providers, administrators, and leaders to facilitate operational and regulatory processes to use and share health data for AI?
d. What are the driving factors for healthcare organizations to provide AI-facilitated health services?
e. Who are the organizational drivers at this organization? Why?
f. What characterizes AI champions/institution leaders promoting use and share of health data for AI?
g. What are the main barriers for institution X to share de-identified health data with the broader community for public good?
h. In the context of public data sharing, what motivates or limit the release of de-identified health data?
i. What enables or disenable institution X from curating and sharing health data for AI research to promote public good?
j. Describe external or internal factors that might influence the data-sharing process at your institution (why or why not)?
k. What influences an organization's decision to work with data curation services? Why or why not?

© 2023 Youssef A, et al. *JAMA Network Open.*
