# Supplementary material for: Organizational Factors in Clinical Data Sharing for Artificial Intelligence in Health Care
Source: JAMA Netw Open. 2023 Dec 19;6(12):e2348422. doi: 10.1001/jamanetworkopen.2023.48422 (PMC10731479; doi:10.1001/jamanetworkopen.2023.48422)
Supplement: Supplement 2. — Data Sharing Statement [file jamanetwopen-e2348422-s002.pdf]

Data Sharing Statement

Youssef. Organizational Factors in Clinical Data Sharing for Artificial Intelligence in Health Care. *JAMA Netw Open*. Published December 19, 2023.
doi:10.1001/jamanetworkopen.2023.48422

Data
Data available: No

Additional Information
Explanation for why data not available: Interview protocol explicitly specified protecting interview participants confidentiality, thus, in this study we did not specify organization names to avoid risk of identifying study participants.
